# Separation and Identification of Highly Efficient Antioxidant Peptides from Eggshell Membrane

**DOI:** 10.3390/antiox8100495

**Published:** 2019-10-18

**Authors:** Qian-Cheng Zhao, Jie-Yuan Zhao, Dong Uk Ahn, Yong-Guo Jin, Xi Huang

**Affiliations:** 1 College of Food Science and Technology, National Research and Development Centre for Egg Processing, Huazhong Agricultural University, No 1 Shizishan Street, Wuhan 430070, China; zhaoqiancheng5696@163.com (Q.-C.Z.); m18202782622_1@163.com (J.-Y.Z.); jinyongguo@mail.hzau.edu.cn (Y.-G.J.); 2 Department of Animal Science, Iowa State University, Ames, IA 50011, USA; duahn@iastate.edu

**Keywords:** eggshell membrane, Na_2_SO_3_-assisted enzymatic hydrolysis, antioxidant peptide, inhibition of liposome peroxidation, mass spectrometry, total antioxidant activity

## Abstract

The enzymatic hydrolysates (EHs) of the eggshell membrane (ESM) were obtained after incubating eggshell membrane in solutions prepared with Na_2_SO_3_ and alkaline protease combinations. The effects of enzyme species, enzyme dosage, Na_2_SO_3_ concentration, and hydrolysis time on the antioxidant activity of the ESM-EH were determined. Also, the correlation between the degree of hydrolysis (DH) and the antioxidant activity of ESM-EH was analyzed. The DH of ESM-EH showed a highly positive correlation with the reducing power (R^2^ = 0.857) and total antioxidant activity (TAA) (R^2^ = 0.876) and performed negative correlation with the Fe^2+^-chelating ability (R^2^ = −0.529). The molecular weight distribution of the ESM-EH was determined by MALDI-TOF/MS. Cation exchange chromatography (Sephadex C-25) was used to isolate the ESM-EH and then the enzymatic hydrolysis fragment (EHF) was obtained. Among the five isolated fragments (F1~F5), fragment 3 (F3), which was composed of 28 polypeptides, showed the highest ability to quench ABTS• (2,2-azinobis-3-ethyl-benzothiazoline-6-sulfonic acid) (90.44%) and also displayed stronger TBARS (thiobarbituric acid– reactive substances) (58.17%) and TAA (303.82 µg /mL) than the ESM-EH. Further analysis of the 28 peptides in F3 identified using LC-MS/MS indicated that five peptides (ESYHLPR, NVIDPPIYAR, MFAEWQPR, LLFAMTKPK, MLKMLPFK) showed high water-solubility, biological activities, and antioxidant characteristics. Finally, the TAA of the synthetic peptide was verified, the synthetic peptides ESYHLPR and MFAEWQPR performed the best activity and have high potentials to be used as antioxidant agents in functional foods, pharmaceuticals, or cosmetics.

## 1. Introduction

The body produces reactive oxygen species (ROS) in normal metabolic activities, ROS including superoxide anions, hydrogen peroxide, hydroxyl radicals, and nitric oxide react with proteins, nucleic acids, lipids, and other biological macromolecules in the body to cause oxidative damage to cells and tissues [1]. Excessive free radicals in the body can cause chronic diseases like diabetes, arthritis, or atherosclerosis, while active antioxidant peptides can inhibit oxidative stress [2], liposomal peroxidation [3], or have the function of scavenging free radicals and significantly inhibit the oxidation of substrate. During the preparation of antioxidant peptides, protein source, enzyme type, and the degree of hydrolysis (DH) affect the activity of the peptides in varying degrees [4]. The amino acid composition, as well as the specific amino acid sequence in peptides, enhances its antioxidant activity. For example, the antioxidant ability of a peptide increases when Pro, His, or Tyr is present in the amino acid sequence, or Val or Leu at the N-terminal position [5]. Many bioactive peptides from food and non-food sources have shown positive health effects [6]. Endogenous opioid peptides, as neurotransmitters, are involved in various physiological activities such as stress response, appetite, depression, etc., and enzymatically produced milk, wheat, meat, etc. produce opioid peptides [7]. Most of the functional polypeptides are prepared using the specific cleavage effect of enzymes to disrupt the secondary structure of the protein, thereby obtaining the target product. For example, protease hydrolysis of oat bran protein is used to prepare antioxidant peptides [8], and milk polypeptide prepared by fungal protease has antibacterial and antioxidant activities [9]. Also, antioxidant and angiotensin-converting enzyme inhibition peptides were obtained from cheese whey [10]. However, due to the presence of abundant structural proteins (keratin), many protein sources such as feathers, hair, and eggshell membrane (ESM) are difficult to hydrolyze using enzymes.

Eggshell membrane (ESM) as a high-quality protein source has received considerable attention to researchers at present. ESM has been clinically shown to relieve joint inflammation [11] and wound healing [12,13]. Due to the acid and alkali resistance of ESM, enzymatic hydrolysis is an effective way to turn it into bioactive peptides with multiple functional properties [14]. The ESM hydrolysates were shown to have free radical scavenging, DNA protecting [15], and skin whitening activities [16], and protect skin from wrinkling [17]. Also, the ESM peptides produced by alcalase and protease S (ALPS) reduced the intestinal oxidative stress in Caco-2 cells [2]. Therefore, the good biocompatibility of ESM polypeptides can be further applied to reduce oxidative stress in living organisms. Previous studies showed that it is difficult to hydrolyze ESM because many structural proteins protect its structure. The disulfide bond in structural proteins is one of the main reasons for its difficulty to hydrolyze [18]. Therefore, it is especially important to find a rapid, green, and efficient hydrolysis method for ESM. Na_2_SO_3_ can reduce disulfide bonds and has been used in food processing and has also been used to extract keratin from feathers [19]. In addition, alkaline proteases can be used for the extraction of polypeptides from animal/plant proteins, such as the extraction of antioxidant peptides from sorghum proteins [20]. So, Na_2_SO_3_ or alkaline protease have the potential to use hydrolyzed ESM. 

At present, the research methods of antioxidant peptides are divided into traditional top-down and emerging bottom-up models. The top-down model means that the antioxidant peptide is obtained by hydrolyzing and separating the protein, and the bottom-up model is the in vitro synthesis of peptides based on characteristic antioxidant amino sequences. The former model is the most common in current research, and the appropriate separation method is chosen according to its nature. Ion exchange chromatography has been widely used in food and fermentation industries because it has many advantages such as strong affinity, mild reaction conditions, and easy recovery in the separation process. Among them, cation exchange chromatography is the most commonly used method for separating enzyme hydrolysate [21,22]. However, combinations of various chromatographic methods such as Sephadex G-25 and G-15 gel chromatography, and SP Sephadex C-25 and Reverse phase high-performance liquid chromatography (RP-HPLC) were also used to separate antioxidant peptides from the gelatin hydrolysate of tilapia skin [23] and rice bran proteins [24]. However, no research on the further purification of eggshell membrane enzymatic hydrolysate (ESM-EH), and structural characterization of the active peptides is not available. 

In this study, Na_2_SO_3_ was used to assist the enzymatic hydrolysis of ESM. Five proteases (alkaline protease, pepsin, chymotrypsin, neutral protease, papain) were tested to seek hydrolysates with the highest antioxidant activity. Then, the antioxidant peptides were isolated from the ESM-EH and identified using MALDI-TOF/MS. Further characterization of the isolated peptide fractions, which included water-solubility, antioxidant activity, and the amino acid sequence was also done to provide a theoretical basis for the potential applications of the peptides in food, pharmaceutical, and cosmetic industries.

## 2. Materials and Methods 

### 2.1. Materials and Chemicals

ESM powder was obtained from Zhongnong Xinghe Biotechnology Co., Ltd (Heilongjiang, China) and alkaline protease (CSA# 9014-01-1) was purchased from Sigma (Missouri, USA). Organic reagents and inorganic salts were from Sinopharm Chemical Reagent Co., Ltd (Shanghai, China). Solutions were formulated with deionized water, and all the chemicals were of analytical grades and used without further purification. 

### 2.2. Preparation and Isolation of ESM-EH Fraction

In order to screen the best treatment conditions to produce ESM-EH and isolate the ESM-EH fractions with high antioxidant activities, single factor experiments with stepwise screening method were conducted: First, 5 proteases (alkaline protease, pepsin, chymotrypsin, neutral protease, papain) at 9 U/mg of enzyme were added with 30 mM of Na_2_SO_3_ and incubated for 4 h. Then, 6 enzyme dosages (0, 3, 6, 9, 12, 15 U/mg) of the selected enzyme with 30 mM of Na_2_SO_3_ were incubated for 6 h to find the best dose for the enzyme. After that, 6 Na_2_SO_3_ concentrations (0, 10, 20, 30, 40, and 50 mM) were tested to determine the best Na_2_SO_3_ concentration. Finally, 5 incubation times (2, 4, 6, 8, 10 h) were tested for the optimal hydrolysis time. For each of the screening steps, 5 g of ESM powder was added to 100 mL of distilled water (50 mg ESM/mL solution) and used to prepare the reaction solutions. The reaction solutions were magnetically stirred for 4 h, added with enzymes and Na_2_SO_3_, adjusted the pH and temperature to the optimal conditions for each protease (Table S1), and then incubated for up to 10 h. At the end of the incubation, the enzyme was inactivated by heating the reaction solution at 80 °C for 20 min and then centrifuged at 5000× g for 10 min. The supernatant was dialyzed for 24 h using a 300 Da intercepted dialysis bag, and then tested for the antioxidant activity by measuring 1,1-diphenyl-2-picrylhydrazyl scavenging activity, Fe^2+^-chelating ability, and reducing power. The best conditions that produce the highest antioxidant activity were selected and used to prepare for the ESM-EH powder to isolate the ESM-EH fractions.

To isolate the ESM-EH fractions, ESM-EH powder was dissolved in acetate buffer (0.02 M, pH = 4) at a concentration of 100 mg/mL and then sequentially filtered through 0.45 and 0.22 μm filters before applying to a Sephadex C-25 cation exchange chromatography (Pharmacia Biotech, Sweden). The separated fractions were collected separately to evaluate the antioxidant activity (Total antioxidant activity, thiobarbituric acid -reactive substances inhibition, 2,2-azinobis-3-ethyl-benzothiazoline-6-sulfonic acid scavenging activity) in the enzymatic hydrolysis fragment (EHF).

### 2.3. Evaluation of the Antioxidant Activity of ESM-EH and EHF

The samples were characterized for antioxidant activity by several different antioxidant indices. Different antioxidant evaluation methods are beneficial to the screening of optimal conditions for peptide preparation [25]. In this work, DPPH•-scavenging activity [26], Fe^2+^-chelation activity [27], and reducing power [28] were used to screen the optimal preparation conditions of ESM-EH. DPPH (1,1-diphenyl-2-picrylhydrazyl) solution (0.1 mM) was equal volume mixed with sample solution (1 mg/mL), then incubated in the dark at room temperature for 30 min. The absorbance at 517 nm was recorded. For the Fe^2+^-chelation assay, 1 mL sample solution was added to 3.8 mL FeCl_2_ ethanol solution and 0.2 mL phenazine. The absorbance at 562 nm was measured after 10 minutes of reaction. The principle of reducing power is that the sample solution reduces potassium ferricyanide to potassium ferrocyanide, and potassium ferrocyanide reacts with ferric chloride to form Prussian blue.

Further, the total antioxidant activity (TAA), the radical scavenging effect, and the liposomal peroxidation inhibition activity of the sample were expressed by the amount of the sample corresponding to the amount of ascorbic acid [29], the 2,2-azinobis-3-ethyl-benzothiazoline-6-sulfonic acid (ABTS) scavenging [30], and thiobarbituric acid– reactive substances (TBARS) inhibition [27]. Brief descriptions of TAA were as followed: 0.1 mL sample solution was added to 5 mL of a blend of ammonium molybdate tetrahydrate (4 mM), sodium phosphate (28 mM), and sulfuric acid (0.6 M). The absorbance at 695 nm was measured after reacted for 90 min in 90 °C. Ascorbic acid solution was used to draw a standard curve equation at 695 nm, and the TAA of the sample is expressed as an amount equivalent to ascorbic acid. For ABTS• scavenging method, 0.1 mL sample solution was reacted with 3.9 mL of ABTS working solution in the dark for 6 min, and then the absorbance was read at 734 nm. Due to the addition of antioxidants in commercial oils and fats, the preparation of liposomes by phospholipids was extracted from egg yolk and chloroform in the TBARS assay. Liposomal peroxidation was initiated by ferric chloride and V_C._

Each experiment was repeated three times, and the deionized water was used instead of sample solution as a blank in all experiments. Both detail steps and equation of antioxidant assays are shown in the Supplementary Materials.

### 2.4. Mass Spectrometry

#### 2.4.1. MALDI-TOF/MS Analysis of ESM-EH

ESM-EH solution (1 μL) was spotted on the sample target, allowed to dry naturally, and then 0.6 μL of supersaturated α-cyano-4-hydroxycinnamic acid was dropped on the samples, naturally dried, and then purged with nitrogen. The wavelength and acceleration voltages were 349 nm and 2 kV, respectively. Data were acquired in positive ion mode and automatic acquisition data mode. The scanning range of primary mass spectrometry (MS) was 99–4011 Ku.

#### 2.4.2. LC-MS/MS Analysis of EHF and Database Research

Desalted EHF aqueous solutions were dissolved in 0.1% formic acid/ 2% acetonitrile and then centrifuged at 4 °C for 10 min. The supernatant was pressure-loaded into a 300 μm i.d. capillary column packed with Acclaim PepMap RSLC C18 to obtain purified peptides for further analysis. Equilibrium A (0.1% formic acid) and Eluent B (0.1% formic acid and 80% acetonitrile) were used as the moving phase. Gradient elution was applied to separate peptides in EHF as following: started with 5% B for 5 min, changed from 5% to 35% B in 20 min, from 35% to 90% B in 5 min, and then ran at 90% B for 5min at a flow rate of 300 nL/min. Full scan mass spectra (350 to 1800 m/z) were acquired in the Orbitrap using an automatic gain control (AGC) target value of 5e4 at a resolution of 17,500. The separated peptides were directly entered into the Thermo Scientific Q Exactive mass spectrometer (Thermofisher, Shanghai applied protein technology co. Ltd) for on-line detection.

The original files of mass spectrometry were processed and converted by MM File Conversion software to obtain the MGF format file, the MS/MS peak list with MASCOT (version 2.5.1 Matrix Science, London, UK) was analyzed and then searched using the Uniprot-gallus database. 

#### 2.4.3. Performance Evaluation of Peptides

The solubility of peptides was calculated using the peptide property calculator [31], and the biological activities of peptides were predicted by PeptideRanker [32,33]. The activity analyses of peptides were done using BIOPEP [34].

### 2.5. Statistical Analysis

All experimental data were presented as mean ± standard deviation (SD), and each set of experimental data was in triplicate. Statistical analysis was performed with SPSS 21.0 (IBM SPSS Statistics, IBM Corp, Somers, NY, USA). Significance and high significance of difference were defined as *p* < 0.05 and *p* < 0.01, respectively.

## 3. Results and Discussion

### 3.1. Effect of Enzymatic Hydrolysis Parameters on the Antioxidant Activity of ESM-EH 

**Figure 1:**
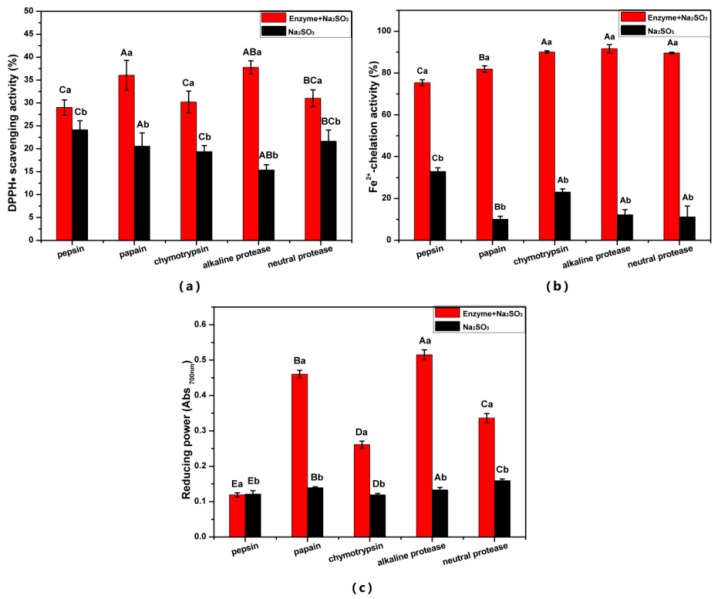
Effects of enzymes species (9 U/mg) on the antioxidant activity of eggshell membrane enzymatic hydrolysate (ESM-EH) with the assistance of 30 mM Na_2_SO_3_. (**a**) DPPH•-scavenging activity; (**b**) Fe^2+^-chelating ability; (**c**) reducing power. Differences in lowercase letters indicate significant differences between enzyme + Na_2_SO_3_ treatment group and Na_2_SO_3_ treatment group (*p* < 0.05), and the difference of capital letters shows the significant difference of enzyme species (*p* < 0.05).

All the Na_2_SO_3_ and enzyme combinations had stronger antioxidant activity than Na_2_SO_3_ alone, indicating that the enzyme plays a key role in producing antioxidant peptides. The DPPH• scavenging ability of the five protease digestion products is shown in Figure 1a. The DPPH• scavenging ability from the alkaline protease treatment group was the strongest (37.76%), followed by the papain treatment group (36.05%); both of them were higher than that of the equivalent sea squirt protein hydrolysate (31%) [35]. The DPPH• scavenging ability of the pepsin group was the weakest (29.00%) in this assay probably because of the acidic conditions that decomposed Na_2_SO_3,_ which weakened the ability of Na_2_SO_3_ to reduce the disulfide bonds. The ESM hydrolysates with chymotrypsin, alkaline protease, and neutral protease showed a good Fe^2+^-chelating power, which was significantly higher (*p* < 0.05) than that of the papain and pepsin treatments (Figure 1b). The ESM-EH from alkaline protease had the strongest Fe^2+^-chelating (91.62%) and iron ion reducing ability, while pepsin treatment showed the lowest ability. However, the Fe^2+^-chelating activity of the five enzyme treatments was stronger than that of the finger millet protein hydrolysate (35.11%) [36] and Pearl millet hydrolysate (22.70%) [37] at the same concentration, indicating that all the ESM-EHs produced have the potential to be used as Fe^2+^-chelating agents. The ESM hydrolysate from the alkaline protease treatment showed the strongest reducing power (0.515), which was significantly higher than that of the other four proteases (*p* < 0.05) (Figure 1c). This is attributed to more active sites of alkaline protease than other enzymes [38]. Therefore, alkaline protease was selected for further optimization.

**Figure 2:**
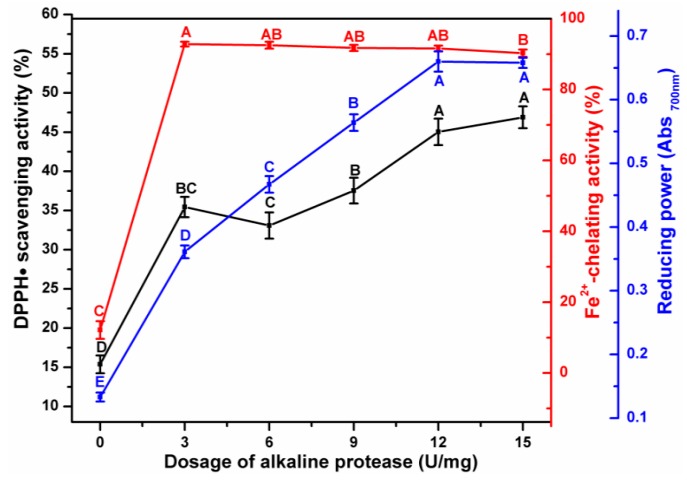
Effect of alkaline protease dosage on antioxidant activity of ESM-EH with the assistance of 30 mM Na_2_SO_3_. DPPH•-scavenging activity (black line); Fe^2+^- chelating ability (red line); reducing power (blue line). The different capital letters indicate significant differences (*p* < 0.05).

As shown in Figure 2, three antioxidant indices showed an increasing trend as the dose of alkaline protease increased. The DPPH•-scavenging activity linearly increased as the dose of the enzyme increased, except for at 3 U/mg dose. The reducing power was also positively correlated with the amounts of enzymes added but reached a maximal value of 0.66 at 12 U/mg. As the dose of the enzyme increased to 15 U, however, it showed an insignificant decrease because of the saturation of binding sites in the substrate [39]. ESM-EH exhibited higher Fe^2+^-chelating activity at low concentration of alkaline protease (3 U/mg), which is due to the reduction of disulfide bonds in the eggshell membrane to sulfhydryl groups. The atoms in the functional groups that form chelates generally include nitrogen, oxygen, and sulfur. The sulfur-containing ligands have a higher affinity for noble metal and heavy metal ions than nitrogen and oxygen-containing ligands [40]. Therefore, 12 U/mg of alkaline protease was selected for further optimization.

**Figure 3:**
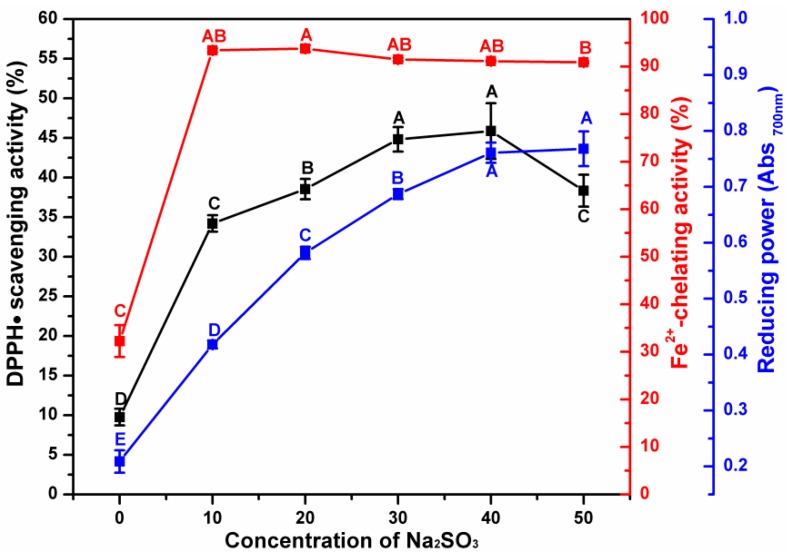
Effect of different concentrations of Na_2_SO_3_ on the antioxidant activity of ESM-EH produced by alkaline protease treatment. DPPH•-scavenging activity (black line); Fe^2+^-chelating ability (red line); reducing power (blue line). The different capital letters indicate significant differences (*p* < 0.05).

The effect of different Na_2_SO_3_ concentrations on the antioxidant activity of ESM-EH is shown in Figure 3. All three antioxidant indices showed a sharp increase when 10 mM of Na_2_SO_3_ was added in the reaction system, indicating the synergistic effect of Na_2_SO_3_ and alkaline protease in producing hydrolysates from ESM. Among the three indices, Fe^2+^-chelating ability was affected the most by the addition of Na_2_SO_3_, especially when the concentration of Na_2_SO_3_ was low (<10 mM). During the enzymatic hydrolysis process, the proteins on the surface of ESM are hydrolyzed first and the disulfide bonds in structural proteins produce a large number of thiol groups. The electron-negative sulfur atoms in the sulfhydryl groups are more conducive to the chelation of ferrous ion. The DPPH•^−^scavenging capacity and the reducing power of the ESH-EH showed logarithmic increases until the concentrations of Na_2_SO_3_ increased to 40 mM. When the concentration of Na_2_SO_3_ was 40 mM, the DPPH•^−^scavenging capacity of ESM-EH reached its maximum (45.87%) but showed a decreasing trend with the increase of Na_2_SO_3_ concentration (>40 mM). The reducing power of ESM-EH reached a plateau at 40 mM of Na_2_SO_3_ with a small increase at a higher concentration (50 mM). Therefore, 40 mM was chosen as the most suitable sodium sulfite concentration.

**Figure 4:**
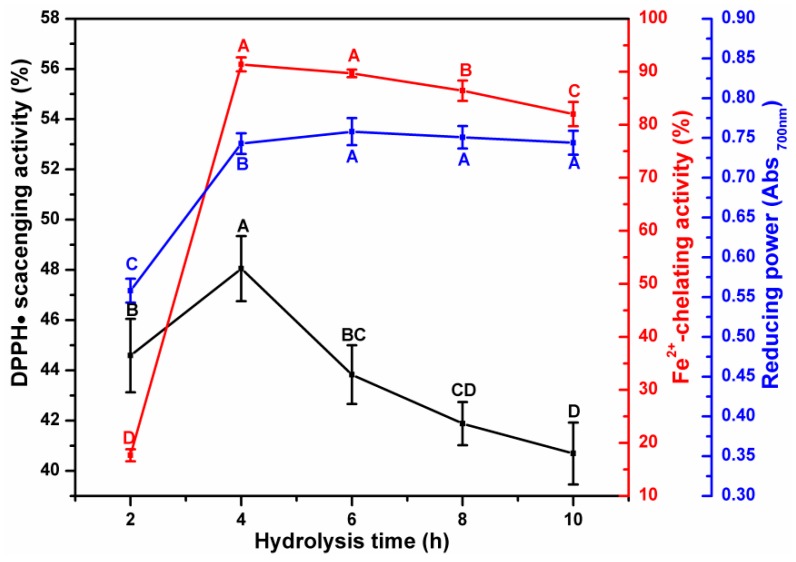
Effect of hydrolysis time (12 U/mg alkaline protease with 40 mM Na_2_SO_3_) on antioxidant activity of ESM-EH. DPPH• scavenging activity (black line); Fe^2+^-chelating ability (red line); reducing power (blue line). The different capital letters indicate significant differences (*p* < 0.05).

The effect of incubation time on the antioxidant activity of ESM-EH indicated that the DPPH•^−^scavenging ability reached the maximum value (48.05%) at 4 h, which was significantly higher than that at 6 h (Figure 4). The highest Fe^2+^-chelating activity was observed at 4 h (91.69%) and was no different from that at 6 h. The reducing power showed a sharp increase as the incubation time increased from 2 h to 4 h. However, the maximum reducing power value (0.758) was reached when the incubation time was 6 h and the further increase of incubation time did not affect the reducing power. It was assumed that excessive hydrolysis could break the sequence of peptides with antioxidant properties [19] due to the non-selectivity of alkaline protease. Hence, 12 U/g, 40 mM Na_2_SO_3,_ and 4 h incubation time were selected as the best conditions for alkaline protease to produce ESM-EH with the highest antioxidant activity.

### 3.2. Characterization of ESM-EH 

#### 3.2.1. Visual and Microscopical Analyses of ESM Membrane During the Enzymatic Hydrolysis Process

**Figure 5:**
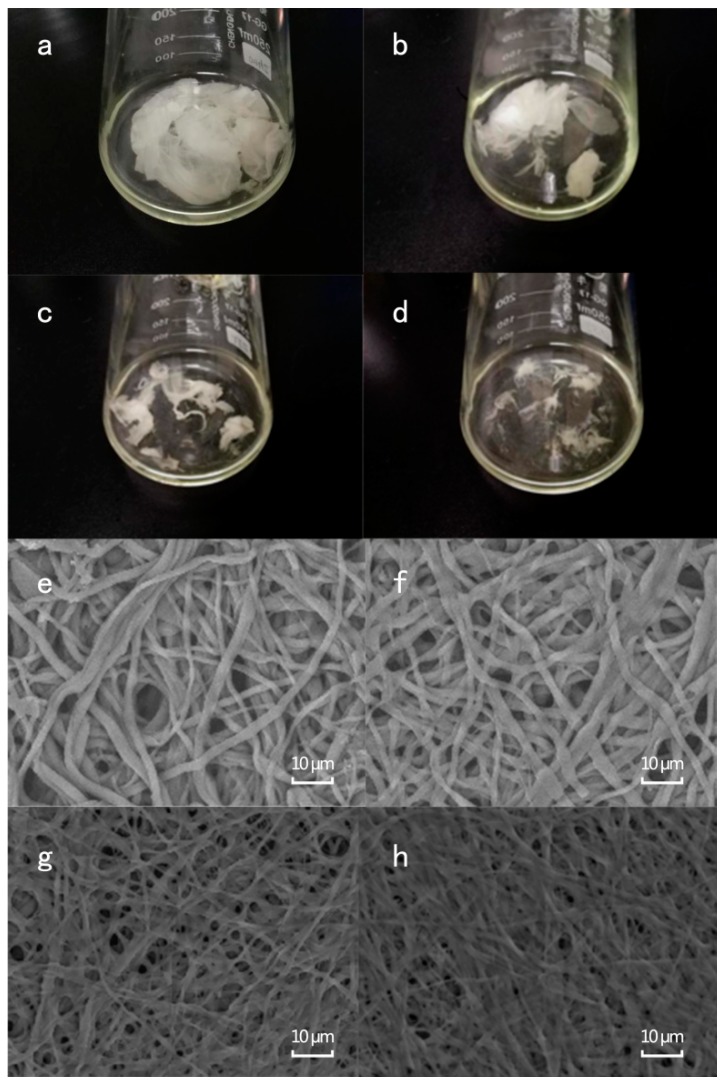
Visual image of alkaline protease and Na_2_SO_3_ combination at different times: (**a**) 0.5 h; (**b**) 1 h; (**c**) 1.5 h; (**d**) 2 h. ESM images of different treatment groups: (**e**) sodium sulfite treatment alone for 4 h; (**f**) alkaline protease alone for 4 h; (**g**,**h**) sodium sulfite + alkaline protease treatment ((**g**) 1 h, (**h**) 1.5 h).

In this process, field emission-scanning electron microscopy (FE-SEM) and visual observations were used to observe the effect of Na_2_SO_3_ and alkaline protease, singly and with their combination on ESM structure. In the visual observation, the ESM treated with alkaline protease and Na_2_SO_3_ combination became thin and transparent after 0.5 h of incubation (Figure 5a). More than 50% of the ESM was dissolved when it was treated with alkaline protease and Na_2_SO_3_ combination for 1 h (Figure 5b). As the incubation time increased to 1.5 h, more of the ESM was hydrolyzed (Figure 5c), leaving only a small amount of ESM residues after 2 h incubation (Figure 5d). The FE-SEM observation showed that the three-dimensional network structure of the native ESM was protected by the stable structural proteins on the fiber surface (Figure S2) [41]. When the ESM was treated with alkaline protease or Na_2_SO_3_ for 4 h, the ESM fibers became thin (Figure 5e,f). However, when the combination of Na_2_SO_3_ and alkaline protease was used, the three-dimensional network structure of ESM was destroyed and became flat (Figure 5g,h). The fiber diameters of the ESM treated with Na_2_SO_3_ and alkaline protease combination was thinner than that treated singly with the alkaline protease or Na_2_SO_3_ for 4 h. In an earlier work, 3-mercaptopropionic acid and acetic acid were used to prepare soluble eggshell membrane proteins [42]. Sodium sulfide is an efficient agent to open disulfide bonds [43] and is greener, water-soluble, and more efficient than other organic reagents in the destruction of disulfide bonds [44]. Also, Na_2_SO_3_ improved the solubility of ESM and assisted the enzymatic hydrolysis of the ESM. The obtained hydrolysates from Na_2_SO_3_ and alkaline protease combination had better antioxidant activity than those from Na_2_SO_3_ or alkaline protease alone.

#### 3.2.2. Analysis of Molecular Weight Distribution of ESM-EH by MALDI-TOF/MS

The MALDI-TOF mass spectrum of ESM-EH obtained by Na_2_SO_3_-assisted enzymatic hydrolysis showed that the enzymatic hydrolysate contained large amounts of low-molecular-weight peptides (Figure S1) (m/z < 881.4). The amount of low molecular weight peptides (m/z < 881.4) in ESM-EH significantly increased, and the high molecular weight peptides (m/z > 1663.8) significantly decreased compared to the extract from the ESM using methanol [45]. This indicated that the Na_2_SO_3_-assisted alkaline protease hydrolysis cut the ESM into small molecular peptides, and the DH was improved. The antioxidant capacity of the peptides in the hydrolysates was related to their molecular weight. The small molecule peptide can react with the free radical more effectively to exhibit better antioxidant activity [37]. Therefore, the small molecular weight of ESM-EH is more conducive to its antioxidant activity.

### 3.3. Analysis of the Correlation between Degree of Hydrolysis and Antioxidant Indices

As shown in Table 1, the DH of ESM-EH showed a strong positive correlation with its reducing power (R^2^ = 0.857**) and TAA (R^2^ = 0.876**), while there was no significant correlation between DH and DPPH•-scavenging activity. The increase of DH allows the ESM protein to be digested into smaller molecule peptides with antioxidant activity [46,47]. Similar to the alkaline protease digestion product of porcine plasma protein [48], the reducing power and DPPH•-scavenging ability increased significantly as the DH increased (*p* < 0.05). The DH of ESM-EH was negatively correlated with the chelating ability of ferrous ions (R^2^ = −0.529*). In a previous study, however, the Fe^2+^-chelating activity of grass carp protein hydrolysate produced with alkaline protease was positively correlated with the DH [49]. The reason for this difference was due to the presence of rich disulfide bonds in the ESM.

### 3.4. Isolation and Identification of EHF in ESM-EH

#### 3.4.1. Isolation and Characterization of EHF

**Figure 6:**
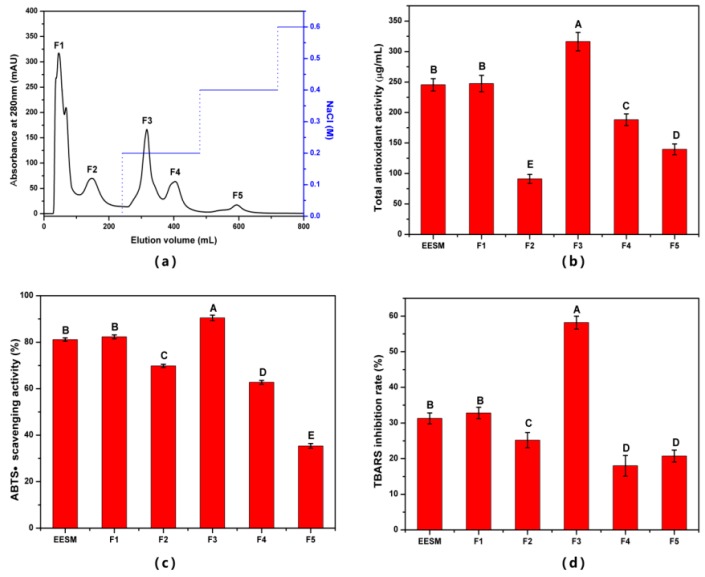
(**a**) Separation of ESM-EH by cation exchange chromatography. (**b**) TAA, (**c**) ABTS• scavenging rate, and (**d**) TBARS inhibition rate of isolated fragments. The different capital letters indicate significant differences (*p* < 0.05).

ESM-EH obtained from sodium sulfite (40 mM) and alkaline protease (12 U/mg) combination with 4 h incubation time was used in this study. Although Sephacryl S-300, Sephacryl S-100 gel Chromatography, Sephadex C-25 cation exchange chromatography, and ultrafiltration were tested to separate peptide fractions from the ESM-EH (Figure S3), the separation effect of Sephadex C-25 was better than others (Figure 6a). The five fragments separated from ESM-EH by cation exchange chromatography were named F1–F5 according to the order of peaks. The total antioxidant activity (TAA), ABTS•-scavenging activity, and liposomal peroxidation inhibitory activity were used to evaluate antioxidant activities of these fractions. 

As shown in Figure 6, the antioxidant activities of F3 were significantly higher than the other four fragments and ESM-EH (*p* < 0.05). The TAA, TBARS inhibition rate, and ABTS•-scavenging rate were 303.82 μg/mL, 58.17%, and 90.44%, respectively. Compared with ESM-EH, the TAA, ABTS•-scavenging rate, and TBARS inhibition rate of F3 increased by 89.22%, 11.50%, and 28.90%, respectively (Figure 6b–d). Among them, the ABTS•-scavenging rate of F3 is higher than GF_B_ (78.61%) which is obtained by separating the pearl millet protein hydrolysate with ultrafiltration and gel column Sephadex G-25 [37]. Therefore, F3 is considered to be the best EHF obtained. The TAA of F3 was higher than that of the antioxidant peptides obtained from *Scomberomorus niphonius* using a continuous microwave-assisted method [29]. Although there is no other research on the inhibition of liposome peroxidation by ESM, F3 showed a strong inhibitory effect on the peroxidation of liposomes. This index indicated a high prospect of F3 as an antioxidant in the food industry.

#### 3.4.2. Analysis of Amino Acid Sequence and Antioxidant Characteristics of F3 

Among the five fragments, F3 showed the highest activities for all three assays (TAA, ABTS•, TBARS). Based on the LC-MS/MS analysis of F3, 28 peptides were identified (Table 2). Further searching for the source protein of the peptide in Uniprot, 22 peptides were matched with the proteins in the *Gallus gallus* (chicken) database (Table 2). It is interesting to note that six peptides were not found in the *Gallus gallus* (chicken) database, which may be attributed to the protein-difference between egg and chicken. The identified antioxidant-active peptides are mostly composed of 2–20 amino acids, usually having a molecular weight of less than 6 Ku [27], which is similar to the results of this work. The peptides 1–22 (Table 2) had good water solubility, indicating the good solubility of F3. The predicted activity score of eight peptides was higher than 0.50. The amino acid sequence of the peptide and the nature of the amino acid side chain influence their antioxidant activity [50]. For example, N-terminal lysine (L) is highly reactive with DPPH• [51], the peptide 15, and 26 in F3 possess this feature. Peptides with 2–3 specific amino acids (S, G, P, and V) next to each other are more active than peptides with only one specific amino acid [52], and the peptides 7, 17, 23, and 24 in Table 2 meet the above requirements. Also, peptides containing A, G, P, V, and L in the amino acid sequence usually have a strong antioxidant activity [24]. Most of the peptides in F3 had this feature. Some amino acids like acidic amino acid residues (D and E) can contribute to antioxidant activities of peptides because they can chelate metal ions through the carboxyl group in the side chain [53], and the peptides 1–11 of F3 (Table 2) have this characteristic. The presence of aromatic residues (Y, W, and F), imidazolyl group (H), and sulfur-containing groups (M and C) can quench free radicals by direct transfer of electrons [54]. Thus, peptides 4–17 and 25–28 (Table 2) have the potential for free radical scavenging activities. Hydrophobic residues (V, L, I, A, F, K) at the C-terminus or N-terminus of the peptide chain enhance the antioxidant activity of peptides [55]. Thus, peptides 1, 5, 10, 15, and 16 in F3 (Table 2) have this property. Moreover, the presence of leucine (L) in the third amino acid position on the left side of the C-terminus (peptide 3, 6, 14) enhances the antioxidant activity [56] (Table 2). According to the comprehensive analysis of water solubility, predicted activity scores, and antioxidant characteristic sequences of the peptides, peptides 6–8, 15, and 16 (ESYHLPR, NVIDPPIYAR, MFAEWQPR, LLFAMTKPK, and MLKMLPFK) are the most promising antioxidant peptides among the 28 peptides from F3.

#### 3.4.3. In Vitro Synthesis and Antioxidant Activity Verification of Peptides 

**Figure 7:**
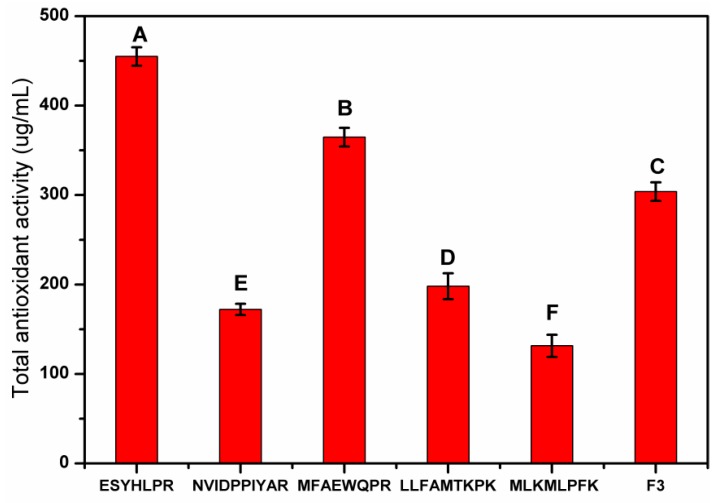
Determination of total antioxidant activity of five predicted peptides. The different capital letters indicate significant differences (*p* < 0.05).

To verify antioxidant activity, five peptides were synthesized in vitro based on their amino acid sequences. The result of TAA evaluation showed that the five synthetic peptides showed acceptable antioxidant activity (Figure 7), in which peptide 6 (454.89 μg/mL) and 8 (364.73 μg/mL) showed stronger antioxidant activity than F3. The mechanism may be due to the presence of free radical scavenger amino acids and the presence of acid residues. The reason for the lower TAA of peptide 7 may be attributed to the presence of the Pro-Pro (PP) structure. Interestingly, peptide 6 has the strongest antioxidant activity, probably due to the lead role of lysine (L) at the third position on the left side of the C-terminus. In general, the peptides with ESYHLPR and MFAEWQPR sequences exhibited the best antioxidant activity and all five peptides shows good antioxidant potential.

## 4. Conclusions

The ESM-EH prepared using the Na_2_SO_3_-assisted alkaline protease under optimal conditions produced peptides with lower molecular weight distribution (m/z <881.4) and showed excellent antioxidant activities. The DH of ESM-EH was positively correlated with the reducing power and total antioxidant activity but had a negative correlation with Fe^2+^-chelating activity. One of the fractions (F3) separated from the ESM-EH using cation exchange chromatography showed a high antioxidant effect: high TAA, ABTS•-scavenging, and liposome oxidation inhibition ability. The five peptides (ESYHLPR, NVIDPPIYAR, MFAEWQPR, LLFAMTKPK, and MLKMLPFK) identified from F3 showed better antioxidant properties. In the further verification of total antioxidant activity, synthetic peptide ESYHLPR and MFAEWQPR performed the best activity, and have high potentials to be used as antioxidant agents in functional foods, pharmaceuticals, or cosmetics.

## Figures and Tables

**Table 1:** Correlation analysis between degree of hydrolysis (DH) and antioxidant activity of ESM-EH.

Index	DPPH• Scavenging	Fe^2+^-Chelation	Reducing Power	TAA
DH	0.465	−0.529 *	0.857 **	0.876 **
DPPH• scavenging	-	−0.625 *	0.827 **	0.573 *
Fe^2+^-chelation	-	-	−0.686 **	–0.471
Reducing power	-	-	-	0.832 **

* Indicates significant (*p* < 0.05) and ** indicates highly significant (*p* < 0.01). TAA: total antioxidant activity.

**Table 2:** Antioxidant peptide amino acid sequences from F3.

No.	Peptide Amino Acid Sequence	Molecular Weight (Da)	Abundance	Water Solubility	Predicted Activity Score	Features of Anti-Oxidation	*Gallus Gallus* (Chicken)
1	KDQLTPSPR	1040.6	1.72 × 10^8^	+	0.33	N-terminal (K). Intrinsic antioxidant activity (P L). Acid residues (D).	+
2	VEPKSPR	811.5	3.47 × 10^7^	+	0.19	Intrinsic antioxidant activity (P V). Acid residues (E).	+
3	VEVYLPR	874.5	7.82 × 10^7^	+	0.27	Intrinsic antioxidant activity (V L). The third amino acid to the left of the C-terminus (L). Acid residues (E).	+
4	RFRWSER	1035.5	4.16 × 10^9^	+	0.55	Free radical scavenger (W). Acid residues (E).	+
5	MHSHIDMK	997.4	1.64 × 10^8^	+	0.43	Free radical scavenger (H W M). C-terminal (K). Acid residues(D).	+
6	ESYHLPR	900.4	7.26 × 10^7^	+	0.52	Free radical scavenger (Y H). Intrinsic antioxidant activity (P L). Acid residues (E). The third amino acid to the left of the C-terminus (L).	−
7	NVIDPPIYAR	1156.6	5.46 × 10^7^	+	0.42	Free radical scavenger (Y). Acid residues (D). Intrinsic antioxidant activity (A P V). Repeat specific amino acids in adjacent positions (PP).	+
8	MFAEWQPR	1063.5	8.35 × 10^6^	+	0.69	Free radical scavenger (M W F). Intrinsic antioxidant activity (P V). Acid residues (E).	+
9	MATWRRDGR	1147.6	8.62 × 10^6^	+	0.40	Free radical scavenger (M W). Acid residues (D). Intrinsic antioxidant activity (A G).	+
10	MITLTELK	947.5	6.70 × 10^6^	+	0.09	C-terminal (K). Free radical scavenger (M). Acid residues (E).	+
11	MEAAMGR	764.3	3.18 × 10^5^	+	0.35	Free radical scavenger (M). Acid residues (E).	+
12	MQALSPR	801.4	5.52 × 10^7^	+	0.34	Free radical scavenger (M). Intrinsic antioxidant activity (A P L).	−
13	RVWHKGR	937.5	1.91 × 10^7^	+	0.43	Intrinsic antioxidant activity (V G). Free radical scavenger (H W).	−
14	MVGSKLPR	886.5	1.11 × 10^7^	+	0.36	Intrinsic antioxidant activity (P V G L). Free radical scavenger (M). The third amino acid to the left of the C-terminus (L).	+
15	LLFAMTKPK	1047.6	1.24 × 10^8^	+	0.33	Intrinsic antioxidant activity (A P L). C-terminal (K). N-terminal (L). Free radical scavenger(M).	+
16	MLKMLPFK	1006.6	4.92 × 10^6^	+	0.74	C-terminal (K). Free radical scavenger (M F). Intrinsic antioxidant activity (P L).	+
17	ETLMGGPLR	972.5	4.14 × 10^6^	+	0.31	Free radical scavenger (M). Intrinsic antioxidant activity (P L G). Acid residues (E). Repeat specific amino acids in adjacent positions (G G).	+
18	GTLHIQK	795.5	1.39 × 10^8^	+	0.14	C-terminal (K).	+
19	GVLSPGR	684.4	1.10 × 10^8^	+	0.40	Intrinsic antioxidant activity (G P V L).	+
20	SALPSPR	726.4	2.97 × 10^8^	+	0.64	Intrinsic antioxidant activity (A P L).	−
21	PVSVAPR	724.4	1.97 × 10^8^	+	0.29	Intrinsic antioxidant activity (A P V).	+
22	QQPVSPR	810.4	5.95 × 10^7^	+	0.37	Intrinsic antioxidant activity (P V).	+
23	MMGGALQPR	959.5	9.05 × 10^6^	−	0.38	Repeat specific amino acids in adjacent positions (GG). Intrinsic antioxidant activity (G A L P).	−
24	IPPVAVR	750.5	2.56 × 10^7^	−	0.29	Repeat specific amino acids in adjacent positions (PP). N-terminal (I).	+
25	MALLLPWK	970.6	2.15 × 10^7^	−	0.81	Free radical scavenger (M Y). C-terminal (K)	−
26	LGRVMYSMANCLLMMK	1916.9	4.73 × 10^7^	−	0.70	Free radical scavenger (C M Y). Intrinsic antioxidant activity (G V A L). C-terminal (K). N-terminal (L).	+
27	MAHVQHLK	962.5	2.69 × 10^7^	−	0.23	Free radical scavenger (M H). C-terminal (K), Intrinsic antioxidant activity (A L).	+
28	MISFCVMK	1014.5	1.34 × 10^7^	−	0.67	C-terminal (K). Free radical scavenger (M F C).	+

The amino acid sequence of F3 obtained by further purifying and separating the ESM-EH. “+” indicates a certain or presence, “−” indicates a negative or nonexistent.

## Supplementary Materials

The following are available online at https://www.mdpi.com/2076-3921/8/10/495/s1, Figure S1: MALDI-TOF/MS of ESM-EH, Figure S2: FE-SEM image of natural eggshell membrane, Figure S3: Tricine-SDS-PAGE of ESM-EH after (**a**) ultrafiltration separated, using the method of (**b**) Sephacryl S-300 and (**c**) Sephacryl S-100 to separate the ESM-EH, Table S1: The optimum reaction conditions of five enzymes for hydrolysis.
