# Supplementary material for: Separation and Identification of Highly Efficient Antioxidant Peptides from Eggshell Membrane
Source: Antioxidants (Basel). 2019 Oct 18;8(10):495. doi: 10.3390/antiox8100495 (PMC6826681; doi:10.3390/antiox8100495)
Supplement: Supplementary file 1 [file antioxidants-08-00495-s001.pdf]

Antioxidant activity assay

1. Total antioxidant activity(TAA)

A slight improvement was made concerning Huang's method . 0.1 mL of 3 mg/mL ESM-EH or EHF was added to 5 mL of a blend of ammonium molybdate tetrahydrate (4 mM), sodium phosphate (28 mM) and sulfuric acid (0.6 M). The mixture was incubated at 95℃ for 90 min, then cooled to room temperature and measured the absorbance at 695 nm. The sample solution was replaced with deionized water as a blank. Ascorbic acid solution (0, 100, 200, 300, 400 μg/mL) was used as the positive control. The TAA of ESM-EH or EHF is expressed as an amount equivalent to ascorbic acid.The resulting standard curve equation is: Y = 0.0011 X + 0.0082. Where R2=0.9986 indicates a high fitness.

2. Free radical scavenging activity

The DPPH• scavenging assay was modified from reported procedures . 2 mL ESM-EH aqueous solution (1 mg/mL) was mixed with 2 mL 1,1-diphenyl-2-picrylhydrazyl radical solution (0.1 mM) which dissolved in 95% ethanol, then incubated in the dark at room temperature for 30 min. The absorbance at 517 nm was recorded. Deionized water instead of the sample as a blank.

The ABTS• scavenging assay was performed essentially as described elsewhere . Briefly, 2.45 mM potassium persulfate and 7 mM ABTS were reacted at room temperature and protected from light for 12-16 h to form the ABTS radical stock solution. The stock solution was diluted with PBS (0.1 M pH 7.4), giving the absorbance of 0.70±0.02 at 734 nm (working solution). 0.1 mL of 1 mg/mL ESM-EH or EHF was mixed with 3.9 mL of ABTS working solution, and kept in dark for 6 min, and then the absorbance was read at 734 nm. Record the absorbance value with an equal volume of deionized water as a blank control.

The DPPH• and ABTS• scavenging activity can be calculated using the following equation(1):

$$\text{Scavenging} \quad \text{activity} \quad (\%) = \frac{A_{\text{blank}} - A_{\text{sample}}}{A_{\text{blank}}} \times 100\ \%$$

3. Fe^{2+}-chelating activity

Partial revision according to previous method . 1 mL of the ESM-EH solution (1 mg/mL) was added to a mixture of 3.7 mL ethanol and 0.1 mL $FeCl_2$ (2 mM), and then 0.2 mL of phenazine was added. The absorbance at 562 nm was measured after 10 minutes of reaction. Blank group uses deionized water instead of sample. The activity was calculated based on the change in absorbance using the following formula(2):

$$Fe^{2+}\text{-chelating activity } (\%) = \frac{A_{\text{blank}} - A_{\text{sample}}}{A_{\text{blank}}} \times 100\%$$

4. TBARS assay

The TBARS assay was used to measure the liposomal peroxidation inhibition activity of samples. Because of the addition of antioxidants in commercial oils and fats, the preparation of liposomes by phospholipids extracted from egg yolk and chloroform , 2 mL of 3 mg/mL ESM-EH and EHF were mixed with 10 mL of liposome emulsion, respectively, and the blank was replaced with distilled water instead of the sample to be tested. Liposomal peroxidation was initiated by 0.1 mL $FeCl_3$ (100 μM) and 0.1 mL V_C (2 mM). The reaction system was incubated at 37 ℃ for 16 h. 2 mL of the reaction solution was added to 2 mL of TCA/TBA reagent, and then heated at 90 ℃ for 15 min. After cooled, the supernatant was measured for absorbance at 531 nm. The standard curve was made with 1,1,3,3-tetraethoxypropane and the TBARS value was expressed as mg(MDA)/ mL(sample). Inhibition of liposome peroxidation capacity using TBARS inhibition rate indicates(3):

$$\text{TBARS inhibition rate}\,(\%) = \frac{A_{blank} - A_{sample}}{A_{blank}} \times 100\%$$

5. Determination of reducing power

Reducing power was measured according to the method of Yildirim . Briefly, 1 mL ESM-EH (3 mg/mL) was added to 5 mL potassium ferricyanide solution prepared by 0.1 M PBS (0.5% w/v) at pH 6.6, and incubated at 50℃ for 20 min. After cooling, 2.5 mL of 10% trichloroacetic acid (TCA) solution was added, mixed uniformly, and centrifuged for 10 min (3000×g). 2.5mL supernatant was diluted 2 times and mixed with 0.5 mL ferric chloride (1% w/v), mix well and react at room temperature for 10 min. Record the absorbance at 700 nm to indicate Reducing force.

Degree of hydrolysis

The free amino nitrogen content is determined by the national standard GB/T 5009.39-2003 amino nitrogen formaldehyde value method. Determination of protein in foods using the national standard of the People's Republic of China GB 5009.5-2016 Kjeldahl method for determination of total nitrogen content.

$$DH(\%) = \frac{\text{Free nitrogen content}}{\text{Total nitrogen content}} \times 100\%$$

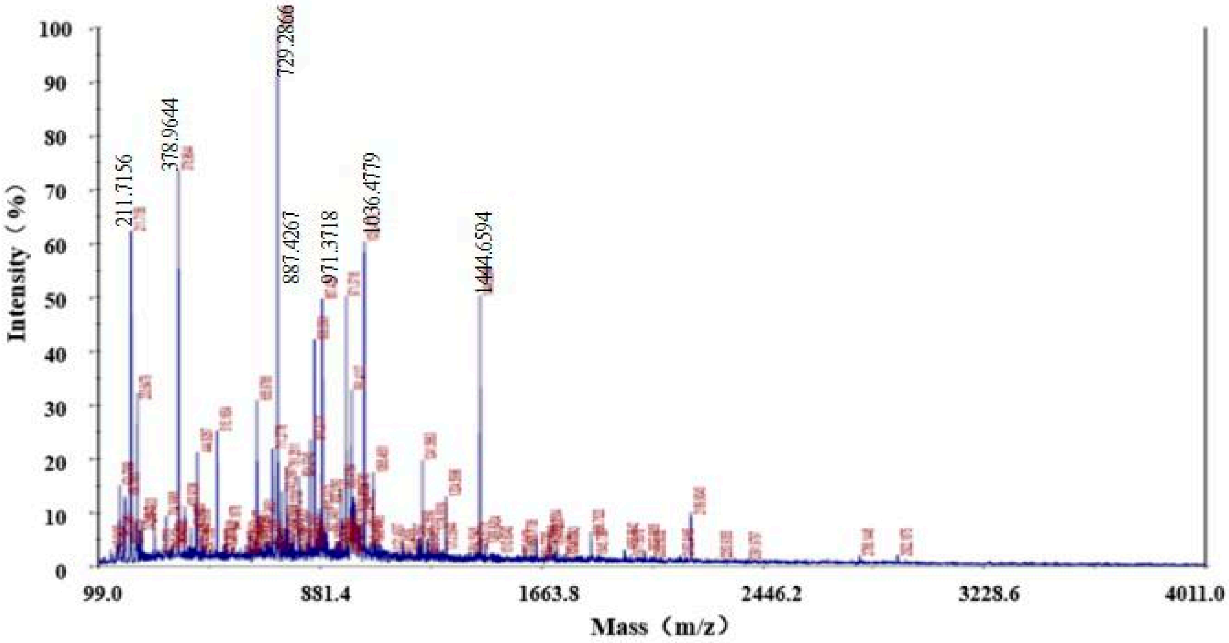

Figure S1. MALDI-TOF first-order mass spectrum of ESM-EH.

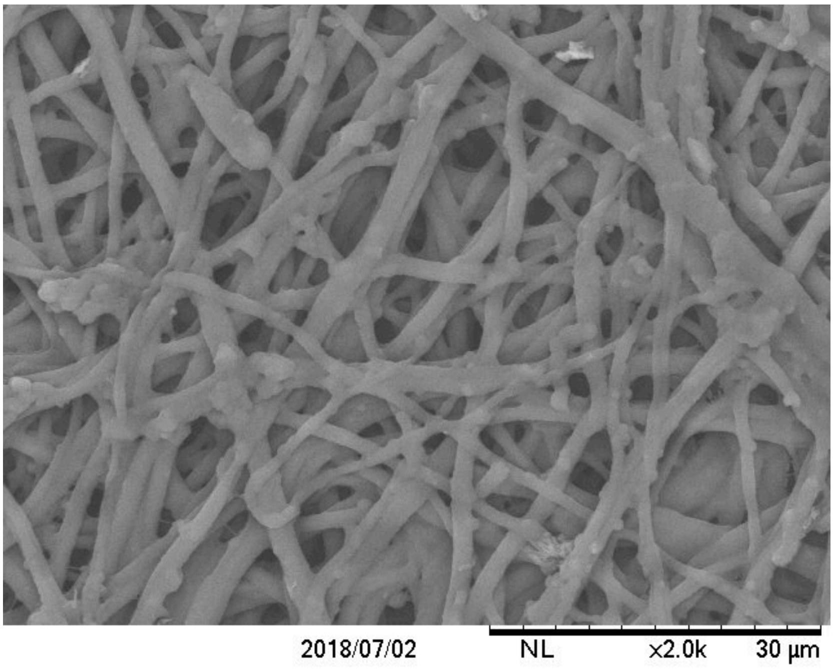

Figure S2. SEM image of native eggshell membrane.

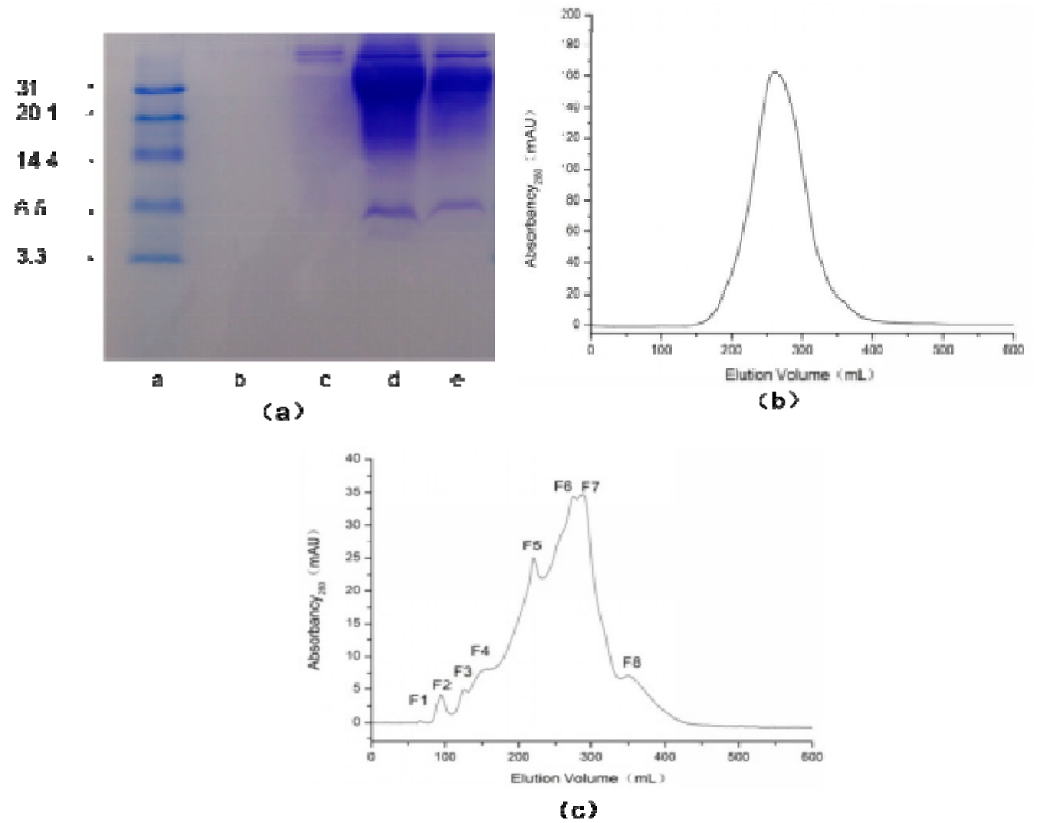

Figure S3. Tricine-SDS-PAGE for separation of products by ultrafiltration (a); Sephacryl S-300(b); Sephacryl S-100(c).

Table S1. The optimum reaction conditions of emzymes for hydrolysis.

Enzyme	pH	Temperature（℃）
Pepsin	2.0	37
papain	6.0	60
Chymotrypsin	7.9	37
Alkaline protease	8.0	55
Neutral protease	7.5	45
